# A Tale of Two Cities: COVID-19 and the Emotional Well-Being of Student-Athletes Using Natural Language Processing

**DOI:** 10.3389/fspor.2021.710289

**Published:** 2021-08-25

**Authors:** Carter Floyd, Susmit S. Gulavani, James Du, Amy C. H. Kim, Jason Pappas

**Affiliations:** The Department of Sport Management, College of Education, Florida State University, Tallahassee, FL, United States

**Keywords:** machine learning, natural language processing, student-athletes, emotional well-being, sentiment analysis, COVID-19

## Abstract

Student-athletes at the Division I institutions face a slew of challenges and stressors that can have negative impacts in eliciting different emotional responses during the COVID-19 pandemic. We employed machine-learning-based natural language processing techniques to analyze the user-generated content posted on Twitter of Atlantic Coast Conference (ACC) student-athletes to study changes in their sentiment as it relates to the COVID-19 crisis, major societal events, and policy decisions. Our analysis found that positive sentiment slightly outweighed negative sentiment overall, but that there was a noticeable uptick in negative sentiment in May and June 2020 in conjunction with the Black Lives Matter protests. The most commonly expressed emotions by these athletes were joy, trust, anticipation, and fear, suggesting that they used social media as an outlet to share primarily optimistic sentiments, while still publicly expressing strong negative sentiments like fear and trepidation about the pandemic and other important contemporary events. Athletic administrators, ACC coaches, support staff, and other professionals can use findings like these to guide sound, evidence-based decision-making and to better track and promote the emotional wellness of student-athletes.

## Introduction

Over the year of 2020, a number of challenges and stressors, such as the public health crisis of COVID-19, nationwide racial justice protests in response to acts of police violence and brutality, and psychosocial isolation imposed by mandatory public health restrictions have emerged that could have a severe negative impact on the psychological well-being of collegiate student-athletes. These challenges and stressors can lead to heightened feelings of anger, anxiety, or fear (McGuine et al., 2021; Shepherd et al., 2021).

According to Facer-Childs et al. (2021), 54.3% of athletes have indicated that they experienced disrupted mental health due to the COVID-19 pandemic, whereas 63.6% indicated disrupted mood or sentiment. A recent narrative review also indicated that athletes are at a heightened risk of adverse mental health symptoms relative to the general population (Reardon et al., 2021). Due to these mental health risks, it is crucial that athletic administrators be able to efficiently diagnose indicators of heightened distress, fear, or anxiety. The current work provides a valuable methodological foundation for day-to-day evaluations of emotional disposition on a broad scale, with thousands of athletes posting publicly on social media every day using natural language processing techniques (NLP). By implementing data scraping and machine-learning-based NLP, athletic administrators can quantify the sentiment of the social media posts of the student-athletes. By mapping changes in these sentiment values over time while comparing the trends against important or impactful events, it is possible to extrapolate a timeline of sentiment affectation. That is, we can pinpoint specific time periods and specific events that occurred during these time periods that might have caused a heightened emotional reaction in student-athletes. Athletic administrators, ACC coaches, support staff, and other professionals can use findings to guide sound, evidence-based decision-making and to better track and promote the emotional wellness of student-athletes.

As a subfield of natural language processing, sentiment analysis, and related text mining techniques automatically categorize the sentiment expressed in a text presented in various forms (e.g., textual information, emojis) posted on social media platforms and online retailing channels. Sentiment analysis techniques have been adopted in various academic disciplines, including public health and medicine. By employing these techniques, researchers have found a close relationship between the sentiment and the mental health and emotional well-being of an individual. For instance, Zunic et al. (2020) found that narratives within online communities formed around the health conditions, such as mental health problems or eating disorders, of an individual have been studied frequently using sentimental analysis to notice any mental health-related symptoms such as suicidal symptoms of the patients.

Furthermore, by applying an emotional decomposition matrix to data scraped from user-generated content on the social media profiles of the student-athletes, we decomposed the attitudes and feelings expressed within social media posts into a set of eight different emotions, which are as follows: anger, anticipation, disgust, fear, joy, sadness, surprise, and trust. This decomposition matrix can determine if user-generated content expresses more than one of these emotions simultaneously. Anecdotally, a student-athlete might express both fear and anticipation before a major change to conference or school athletic policy is announced.

There are numerous practical applications of a real-time analytical tool for sentiment evaluation. They could be used to tailor or guide organizational policy-making (Kowalski et al., 2020) to target specific athletes or groups of athletes (e.g., athletes of a particular sex or in a particular sport who are exhibiting heightened negative sentiment) for aid, or to anticipate and project future changes in sentiment. More specifically, collegiate athletic organizations like the Atlantic Coast Conference (ACC) can use the sentiment analysis techniques, proposed herein, to monitor expressions of the athletes regarding the feelings of being socially and emotionally supported, by firstly monitoring the overall sentiment trends and then implementing outreach intervention programs to mitigate the lingering impact of adverse mental health outcomes and promoting the optimal functioning under the paradigm of positive psychology (Seligman, 2012). Athletes who express a lack of support have demonstrated heightened academic anxiety (Li et al., 2021) and degradation of athletic identity, or the “strength with which people identify with and embrace their role as an athlete” (Graupensperger et al., 2020). Additionally, research has found links between heightened feelings of anxiety and decreased athletic performance (Khan et al., 2017); so it is vital from both an ethical and a business standpoint that collegiate athletic organizations be able to fully understand the sentiments of their constituent athletes.

Although the current body of research has adeptly determined the connection between feelings of social support and academic and athletic outcomes for student-athletes, thus far no researchers have proposed a methodological approach that can assess such feelings for thousands of student-athletes in real time. This work, while limited in scope, offers a methodological foundation that could be expanded to assess fluctuations and trends in the sentiment of the athlete.

In sum, we apply two machine-learning-based NLP techniques, tripartite sentiment analysis through a logit regression classifier and an emotional decomposition-based machine learning algorithm, to the data generated by ACC student-athletes from March 2020 through February 2021 to evaluate the composition of and changes in student-athlete sentiments during the COVID-19 pandemic.

## Methods

### Data Collection

The data were scraped from the Twitter accounts of ACC student-athletes from four different sports—football, women's volleyball, and men's and women's basketball— across four different ACC member institutions. These sports were selected to ensure a balanced sample in terms of both athlete gender and the season of play (i.e., fall, winter, and spring), as well as based on their financial and reputational significance to the ACC. Considering the fact that the sport leagues and NCAA suspended play due to the spike of COVID-19 cases across the United States (NCAA, 2020), the timeframe for the data collection was from March 2020 until February 2021 to examine the change of sentiments and emotions during the pandemic times.

We scraped and retrieved a total of 2,649 tweets from twitter.com by utilizing both the Twitter streaming API with Tweepy and Octoparse 8.1. The extracted tweets consisted of raw text data from the official Twitter accounts of student-athletes. With each line of raw tweets, we extracted and retained the original textual contents, time posted, user handle, number of views, retweets, and likes published on the official Twitter accounts of the athletes.

### Text Mining

We incorporated a data preprocessing workflow used by sport management scholars to remove unusable or irrelevant information prior to the main analyses (Davidson et al., 2020). In this process, we eliminated extra white spaces, transformed textual contents to lower cases, removed numbers and special symbols (e.g., hyperlinks), deleted punctuations, and removed some common English stop words. These stop words refer to a list of words that convey little value and meaning to analyze the sentiments of the people, and are intended primarily for syntax construction and flow. Examples include personal pronouns and their variants, as well as major prepositions. Further, a corpus was created for the data by using the TM (Feinerer et al., 2008) and SnowballC packages (Bouchet-Valat, 2020). Also, the procedure of stemming and lemmatization was incorporated to group the transformed forms of a word so that they can be analyzed as a single item, identified by the dictionary form of the word. Once a clear corpus was created, we developed a document-term matrix to facilitate the subsequent sentiment and analyses (Nave et al., 2018).

### Sentiment Analysis

The sentiment analysis was conducted using two different algorithms. First, we conducted a machine-learning-based tripartite sentiment analysis through a logit regression classifier to produce a score that represented the sentiment of the Twitter text string in Python. Specifically, we capitalized on artificial intelligence to analyze the textual data. VADER is adopted as a sound machine-learning-based natural language processing tool that is precisely tuned to analyzing Twitter data. It constructed a valence-aware sentiment lexicon by combining existing sentiment word banks and a proprietary list of emojis (e.g., “:-”), sentiment-implied acronyms (e.g., “LOL”), and slangs with sentiment value (e.g., “giggly”) (Hutto and Gilbert, 2014). The package returns a decimal between −5 and 5, decoding overall sentiment for each specific string of tweets. To breakdown, −5 indicates extremely negative sentiment and 5 implies extremely positive sentiment, whereas 0 denotes neutrality for the analyzed textual content. To justify the external validity, we used a 70:30 random split to form a twin-sample to validate the results of the clustering derived from the training set on the testing set. The formation of separate training and testing datasets adopts a firewall principle that searches for an optimized loss function from the training dataset as an input. Particularly, the loss function was minimized using data from the training dataset. The function is then applied to the previously-unseen testing data (from the identical distribution as the training set) to evaluate the model performance. The textual data were converted into numerical data before being partitioned into training and testing datasets using the vectorization function of Python. The data were labeled based on the polarity of the sentiment score values generated from the sentiment analysis algorithm for each tweet. The predictive accuracy of the machine-learning model was evaluated by the “confusion matrix” function from the Scikit-learn library.

Next, the work incorporated a decomposition-based machine learning approach to identify the most salient emotions from the data. As a lexicon-based approach, we used the frequency of lexicon words that appeared in a tweet to measure the strength of a specific emotion breakdown with eight emotional themes (i.e., anger, anticipation, disgust, fear, joy, sadness, surprise, and trust). This method has the benefit of relying on the lexicon of the text to evaluate the frequency of commonly used words based on the NRC emotion lexicon algorithm (Rose et al., 2018). We then calculated a compound score to create a data visualization of how the eight emotional sentiments changed each month in relation to the progression of the COVID-19 pandemic and the occurrence of other major societal events over the examined period of time frame.

## Results

The results indicated that more than half (54.92%) of the tweets of the athletes communicated informational messages with a neutral sentiment. Further, 31.1% of the tweets communicated a positive sentiment, whereas only 13.98% of the tweets communicated a negative sentiment. Table 1 provides examples of tweets that were categorized as either neutral, positive, or negative. The results of the confusion matrix for the tripartite sentiment analysis indicated that overall the natural language processing model was able to correctly predict 89.38% of the total cases. Specifically, the model was able to correctly predict 90.47% of negative sentiments and 88.29% of positive sentiments, suggesting an adequate level of predictive accuracy, consistent with the previous empirical work using machine learning (Casanova et al., 2020).

**Table 1:** Examples of positive, negative, and neutral sentiments and the related sentiment scores generated by the tripartite sentiment analysis.

**Types of sentiment**	**Sentiment score**	**Example**
Negative	−3	Michelle Cusseaux was shot and killed by Phoenix police during a mental wellness check. Say her name. https://t.co/jMW2Pld0o9
Positive	3	Today's Mantra: I AM love. You are love. We are love. May all beings awaken to love.
Neutral	0	#DeacsDecide: 99 percent of @WakeForest's 400-plus student-athletes are now registered to vote in either North Carolina or their home state. Are you?

**Figure 1:**
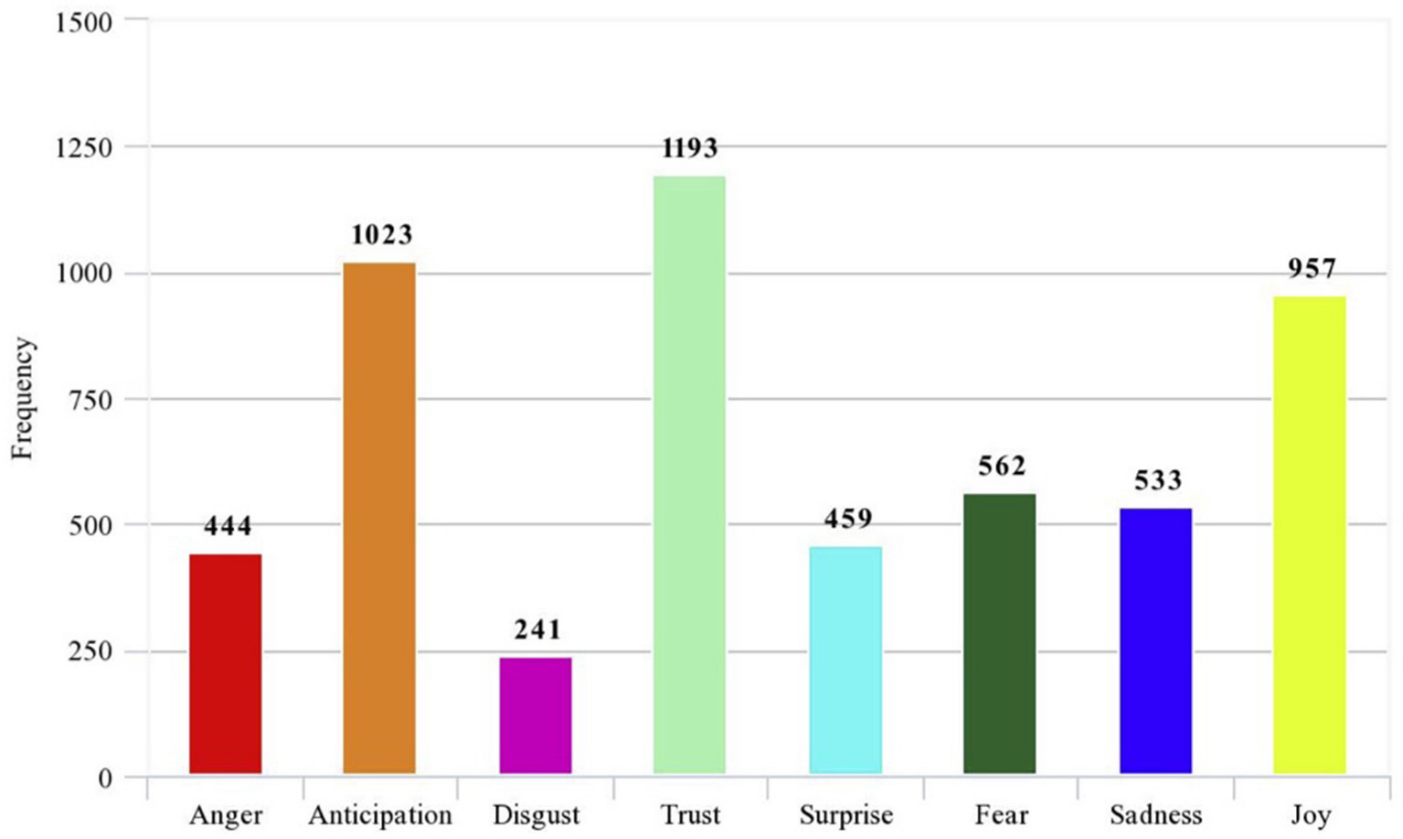
Results of machine-learning-based sentiment decomposition analysis.

Figure 1 displays the results of the emotional decomposition analysis depicting the extent to which the eight emotional themes were embedded in the scrapped Twitter data. Table 2 illustrates examples of the emotions elicited with the associated tweets. It should be noted that the emotions of “trust,” “anticipation” accompanied by “joy,” and “fear” were recognized as top emotions conveyed by the student-athletes during the outbreak. During the global pandemic, student-athletes faced unpredictable uncertainty in terms of their future opportunities in athletics. The postponement and suspension of the games, in particular, heightened the sense of anxiety and distress of student-athletes about the existing situation. Besides, the widespread hope that the college athletics season will begin and end on the schedule had given way to trepidation as teams continued to witness athletes with COVID-19 infections during voluntary practices, and several states had either slowed or reversed certain facets of their reopening plans in response to the rising case numbers.

**Table 2:** Examples of emotions elicited corresponding to respective tweets.

**Tweet**	**Emotions elicited**
Focused improvement on your #CLIMB #EverythingIsConnected	Joy, trust
Proud of these guys for their continued growth in the classroom! #KeepCLIMBing #WORK	Anticipation, joy, trust
We live in a crazy world BE SMART BE SAFE?	Anger, fear, joy, sadness, trust

**Figure 2:**
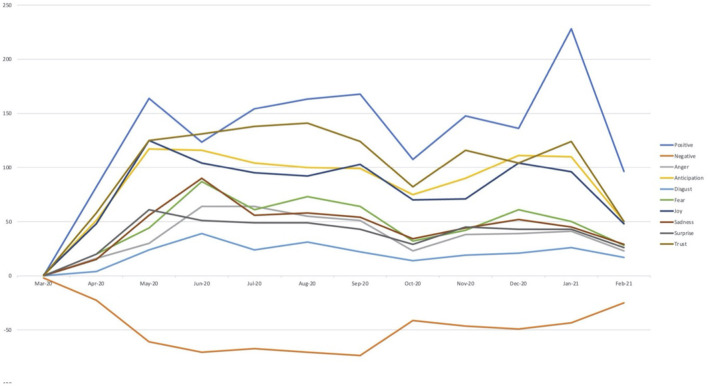
Trend lines depicting the trajectories of decomposed emotions over the examined time frame.

Figure 2 represents the trendline of the sentiments that were elicited throughout the year. The trendline depicts the frequency with which the positive and negative sentiments as well as the eight emotional themes were elicited throughout the year. The figure shows that the positive sentiments peaked in the month of January 2021, which corresponds with the commencement of the vaccination program throughout the country. Also, the positive sentiments were elicited to a greater degree in September 2020, which corresponds to the commencement of the college football season. During this time period, even the emotions of anticipation, trust, and joy were elicited to a greater extent. This indicates that the student-athletes were excited for the start of the college football season and anticipated that the season would unfold positively. Further, the negative sentiments were elicited to a greater extent from June 2020 to September 2020. The murder of George Floyd occurred in the month of May, prompting nationwide “Black Lives Matter” protests. Due to these incidents, negative sentiments were elicited to a greater extent and the emotions of sadness, fear, disgust, and anger also peaked during this time period.

## Discussion

Despite the numerous physiological and psychosocial challenges that these student-athletes were presented with in 2020, big data sentiment analysis found that their social media posts displayed a nearly even mix of positive and negative sentiments, with the four most commonly expressed emotions on Twitter being joy, trust, anticipation, and fear (see Figure 1). Three of these four emotions have positive connotations, suggesting that, at least publicly on social media, these athletes tried to maintain an optimistic attitude. Furthermore, the players showed appreciation for the contributions of both conference administrators and individual colleges in attempting to maintain a semblance of normalcy in their lives. Having a sense of belonging with your peers and having a connection with the university is beneficial for ensuring a sense of psychological well-being (Wann, 2006).

At the same time, the scars of 2020 were evident in both the tripartite sentiment model and the emotional decomposition matrix. As expected, May and June, Figure 2, showed negative sentiments overtaking positive sentiments, a trend that coincides with the resurgence of racial justice protests in response to the newest series of police killings throughout the country. This trend is mirrored with the emotional decomposition matrix, where positive emotions like joy and anticipation became less frequent in May and June, whereas emotions like anger, fear, disgust, and sadness spiked significantly. Negative emotions remained prevalent throughout the summer, coinciding with ongoing protests throughout the country, but began to subside around the end of summer leading into September and October.

Analyzing these sentiment trends against a timeline of major events for the sample of student-athletes, we determined that the decreasing intensity of negative emotions might be connected with the resumption of normally scheduled athletic activity (the ACC announced a comprehensive COVID-19 testing plan on August 18 and the conference football season began on September 3). It could also relate to the increased focus of ACC on speaking out against racial injustice and expanding structural and social support resources for its Black athletes during the 2020–2021 athletic calendar year (Atlantic Coast Conference, 2020). While the conference did not employ the sentiment analysis methods utilized in this work to craft its racial justice initiatives, the changes were made in response to public outcry from the student-athlete population; conceptually, this process was very similar to the sentiment-driven policy-making model we proposed, with the exception that using big data to craft policy would allow an organization to tune its policy decisions more accurately and expediently to the needs of its constituents.

Using text mining and NLP techniques to analyze sentiments allows us not only to extrapolate sentiment trendlines, but to analyze the contents (text) of the tweets themselves to determine popular topics. For example, we conducted a word frequency distribution of the sampled tweets (after removing prepositions, connecting phrases, and other stop words from the dataset) and found that words like “game,” “season,” and “work” were all among those most frequently used by the athletes, suggesting that athletic activity was important to the athletes. Organizations like the ACC could use this data as a way of evaluating the potential shortcomings of their relationship with their constituents. In this case, the trendlines (Figure 2) indicate that conference officials need to do a better job of fostering positive sentiments within their student-athlete population during the summer months, while a content analysis shows specific topics that are important to the athletes.

While acknowledging the merits of the paper, there are several drawbacks associated with it. First, our emotional analysis was based on the individual words that comprised each tweet, but this method does not consider the semantic meaning of the whole tweet, which is much more difficult to quantify given the complexity of lingual expression. We acknowledge that this sentiment lexicon cannot cover the complete domain of knowledge and cannot extract the exact meaning of each word in context. NLP is unable to imply the meaning of textual contents, analyze underlying tone and connotation, and interpret statements accurately without a specific context. For example, in “if I had a dollar for every time my name was pronounced wrongly,” “wrongly” here does not express either the emotion of anger or sadness. Thus, the algorithm does not account for the context specific tweets such as sarcasm or misapplied words. Future studies should investigate beyond the conventional transcript content and capitalize on other forms of social media (e.g., short video sharing via TikTok, Su et al., 2020) through employing recent developments in multimodal sentiment analysis to analyze the complexity of complementary information sources including texts, audios, images, and videos (Soleymani et al., 2017).

Second, our research focused on the tweets from specific student-athletes. We did not analyze tweets from student-athletes competing in Division II or III collegiate athletics. Future research may want to extend our projects to analyze sentiments that are elicited in student-athletes competing in various levels of collegiate athletics, as their priorities could differ significantly from Division I athletes. Third, it remains to be seen if this discrepancy in sentiment between the fall athletic season and the summer offseason is a persistent trend or if it was a one-time occurrence resulting from the unique circumstances of the COVID-19 pandemic (e.g., cancelation of all spring and summer sports, unpredictable schedule alterations, social isolation, the health risks of a once-in-a-lifetime respiratory virus, and a nationwide civil rights movement).

Fourth, it is possible that student-athletes, either voluntarily or at the direction of coaches and administrators at their school, self-censor their thoughts and opinions on social media to avoid institutional or public backlash. The dynamics of public expression of student-athletes through social media are not universal, different programs have different policies (Hopkins et al., 2013, p. 36–41), and these policies are typically established and enforced internally (i.e., coaches and schools do not announce publicly that their athletes are prohibited from discussing certain topics on social media). Additionally, even if athletes are not explicitly prohibited from sharing certain content on social media, fear of negatively impacting intrateam dynamics might lead them to self-censor or refrain from sharing certain ideas.

It should also be noted that the total number of tweets decreased noticeably during the Fall athletic season. As all NCAA sports were canceled over the summer, fall represented the first opportunity for many teams to participate in organized team activities. Additionally, fall marks the beginning of the academic year for these student-athletes as well, and so the decrease in the total tweets could be attributable to the full athletic and academic schedules for the athletes. However, this seasonal decrease in total tweets also raises questions about the ability of athletes to express their genuine emotions in a public forum like Twitter. Some college coaches have instituted team-wide social media bans in the past as a way of avoiding controversy and minimizing the chances of an athlete posting something that a majority of fans might not agree with.

Finally, we wanted to acknowledge that NLP might be prone to human errors and subjective biases in the development of dictionaries and classifiers, although the task of analyzing large-scale textual data would have been labor-intensive to perform using traditional manual approaches. It is straightforward to depict a change in sentiment as an abstract concept with NLP, but it is also important to remember that the trendlines presented in Figure 2 represent genuine emotions expressed by real people. This quantitative, data-driven approach to analyzing sentiments is an effective tool to create a “snapshot” of the emotional consensus of the constituents of an organization, but it can never fully capture the felt emotions and lived experiences of these constituents, which is why even organizations that employ these big data-driven approaches to measuring constituent sentiment should still prioritize facilitating candid, productive discussions (i.e., qualitative research) with those constituents to determine how to address various mutual challenges. In other words, it is still incumbent upon organizing bodies like the ACC to be proactive in diagnosing and addressing problems before they manifest in more extreme or destructive ways, rather than being reactive and waiting until their constituents demonstrate significant negative sentiment to begin troubleshooting and exploring solutions.

## Data Availability Statement

The raw data supporting the conclusions of this article will be made available by the authors, without undue reservation.

## Author Contributions

CF conducted the literature review and data interpretation and drafted the first version of the paper. SG collected and analyzed the data and created data visualizations. JD oversaw the idea generation and manuscript development, guided data scrapping and analysis process, revised and partially rewrote paragraphs in the article, and checked the overall manuscript for coherence, consistency, and format. AK and JP involved in the conceptualization of the manuscript, provided feedback, and helped revise the paper during the manuscript development process. All authors contributed to the article and approved the submitted version.

## Conflict of Interest

The authors declare that the research was conducted in the absence of any commercial or financial relationships that could be construed as a potential conflict of interest.

## Publisher's Note

All claims expressed in this article are solely those of the authors and do not necessarily represent those of their affiliated organizations, or those of the publisher, the editors and the reviewers. Any product that may be evaluated in this article, or claim that may be made by its manufacturer, is not guaranteed or endorsed by the publisher.
